# A Facile Approach to Prepare Black TiO_2_ with Oxygen Vacancy for Enhancing Photocatalytic Activity

**DOI:** 10.3390/nano8040245

**Published:** 2018-04-16

**Authors:** Shihao Chen, Yang Xiao, Yinhai Wang, Zhengfa Hu, Hui Zhao, Wei Xie

**Affiliations:** 1 School of Physics &Optoelectronic Engineering, Guangdong University of Technology, Guangzhou 510006, China; shchenbhx@163.com (S.C.); xiaoyxy1023@163.com (Y.X.); zhfhu@gdut.edu.cn (Z.H.); kkhui@gdut.edu.cn (H.Z.); 2 School of Physics Science and Technology, Lingnan Normal University, Zhanjiang 524048, China; xiewei@lingnan.edu.cn

**Keywords:** photocatalysis, black TiO_2_, oxygen vacancies, photodegradation

## Abstract

Black TiO_2_ has triggered worldwide research interest due to its excellent photocatalytic properties. However, the understanding of its structure–property relationships and a more effective, facile and versatile method to produce it remain great challenges. We have developed a facile approach to synthesize black TiO_2_ nanoparticles with significantly improved light absorption in the visible and infrared regions. The experimental results show that oxygen vacancies are the major factors responsible for black coloration. More importantly, our black TiO_2_ nanoparticles have no Ti^3+^ ions. These oxygen vacancies could introduce localized states in the bandgap and act as trap centers, significantly decreasing the electron–hole recombination. The photocatalytic decomposition of both rhodamine B and methylene blue demonstrated that, under ultraviolet light irradiation, better photocatalytic performance is achieved with our black TiO_2_ nanoparticles than with commercial TiO_2_ nanoparticles.

## 1. Introduction

Since the pioneering work of Fujishima and Honda in 1972 [1], titanium dioxide (TiO_2_) has attracted extensive interest as a widely used semiconductor photocatalyst in the fields of hydrogen production, photocatalytic water-splitting [2], environmental protection technologies [3] and photocatalytic reduction of carbon dioxide [4].Especially in the area of environmental protection, textile dyes and other industrial dye stuffs constitute one of the largest group of organic compounds that represent an increasing environmental danger. Therefore, improving the efficiency of photocatalysis is a current research hotspot. Absorption of light to generate electron-hole pairs and effective charge separation are of great significance for enhancing the efficiency of the photocatalytic reaction in TiO_2_ semiconductor materials. In general, the more light TiO_2_ can absorb, the more excited charges are likely to be on its surface, improving its photocatalytic efficiency [5]. However, the large band gap of anatase TiO_2_ (~3.2 eV) greatly limits its optical absorption in the UV region of the solar spectrum, resulting in poor efficiency for solar-driven photocatalysis.

Therefore, great efforts have been devoted to modifying the band structure to enhance the visible-light absorption, such as doping TiO_2_ with metal [6,7,8,9] or nonmetal [10,11] elements and co-doping with metal and non-metal elements[12,13,14]. All these efforts have enhanced its visible light absorption and photocatalytic activity. However, the results are not yet satisfactory. 

Recently, a hydrogenated black TiO_2_ (B-TiO_2_) material was reported by Mao and co-workers, having a narrow bandgap of ~1.5 eV with increased light harvesting efficiency in the visible and infrared regions and enhanced photocatalytic activity [15]. Unsurprisingly, this discovery has triggered worldwide research interest in black TiO_2_ nanomaterials, which represent a major breakthrough for TiO_2_ in photocatalysis. Since then, different methods for the synthesis of black TiO_2_ have been developed, which have allowed the preparation of hydrogenated or reduced TiO_2_ of different colors like yellow, blue, gray and black, by the use of different initial TiO_2_ materials and reaction conditions [16,17,18]. Most of these approaches can be divided into two types: reduction from TiO_2_ and incomplete oxidation from low-valence-state Ti species. To reduce white TiO_2_ to black TiO_2_, Al, Mg, Zn, H_2_, NaBH_4_, imidazole and ascorbic acid have been used as reducing agents [19,20]. For example, Liu et al. reported black TiO_2_ nanotubes obtained by pressurized H_2_ reduction at high temperature (500 °C, 20 bar, for 1 h), with a high photocatalytic hydrogen production rate [21]. Wang et al. reported a mass-production approach to synthesize black TiO_2_ with a unique crystalline-core amorphous-shell structure (TiO_2_@TiO_2−x_), by using aluminum instead of hydrogen as the reducing agent [22]. Sinhamahapatra et al. developed a controlled magnesiothermic reduction to synthesize reduced black TiO_2_ under a 5% H_2_/Ar atmosphere, with an optimum bandgap and band position, oxygen vacancies, surface defects and charge recombination centers and showing significantly improved optical absorption in the visible and infrared regions [23]. For the second approach, the Ti sources TiH_2_, Ti, TiO, Ti_2_O_3_, TiCl_3_ and TiN have been incompletely oxidized to synthesize black or hydrogenated TiO_2_ [18,24]. For example, Liu et al. synthesized rice-shaped Ti^3+^ self-doped TiO_2−x_ nanoparticles by mild hydrothermal treatment of TiH_2_ in a H_2_O_2_ aqueous solution; the particles showed a strong absorption from the UV to the visible light regions and retained their light-blue color upon storage under ambient atmosphere or water, for one month at 40 °C [25].Pei et al. prepared gray TiO_2−x_ with enhanced visible light photocatalytic activity by a facile hydrothermal treatment of TiO in HCl solution [26].All these synthetic approaches resulted in changes in the TiO_2_ color and enhancement of its light absorption. Surface lattice disorders, high-concentration of oxygen vacancies, Ti^3+^ ions, Ti-OH groups and Ti-H bands have been found in some black TiO_2_ and explain their color change, enhancement of light absorption and photocatalytic activity. These highly defective TiO_2,_ referred to as Ti^3+^ ions/oxygen-vacancy self-doping (TiO_2−x_) and H-doping (TiO_2−x_H_x_), always present black color and possess strong visible light absorption. Some papers have also reported prepared black TiO_2_ that have only oxygen vacancies but no Ti^3+^ ions. For example, Wang et al. suggested the absence of Ti^3+^ in hydrogenated black TiO_2_ nanowires treated at 450 °C [27]. Dong et al. synthesized defective black TiO_2−x_ with a remarkable photocatalytic activity by a facile anodization technique and indicated that there were oxygen vacancies present but no Ti^3+^ [28]. This may be attributed to their lower preparation temperatures (≤450 °C). Furthermore, the defect structure of black TiO_2_ is the major factor that determines its various properties, including color change, light absorption and photocatalytic activity [29]. According to previous papers, black TiO_2_ with disordered shell/hydroxyl groups can induce valence-band/conduction-band tails, that reduce their bandgap [15,30]. They can also induce oxygen vacancies, Ti^3+^ ions and H-doping defects that reduce the bandgap and introduce mid-gap/localized-donor states below the conduction band, in addition to an upshift of the Fermi level [31,32],which renders color changes. For example, for Ti^3+^ self-doping black TiO_2_, the localized excitation of the Ti^3+^ ions via 3d transitions from the gap state to the empty excited state, significantly increases its light absorption and extends it to the visible region [22].

However, some questions still exist regarding black TiO_2_. For example, there is no consensus on what are the most essential modifications and the most effective synthetic method for their preparation. Some black TiO_2_ do not show the expected efficiency in visible light. Furthermore, their preparation is cumbersome and costly. The oxygen vacancies and Ti^3+^ species created during their synthesis are usually not stable enough because the TiO_2_ reduction occurs mainly on its surface. These factors severely limit their wide applicability. Therefore, developing a simple and economic strategy to synthesize a higher stability, higher performance black TiO_2_ photocatalyst is still a great challenge.

Inspired by the above-mentioned considerations, we have developed a facile technique for synthesizing black TiO_2_ nanoparticles, for the first time by low-temperature annealing in a vacuum-tube furnace. The obtained samples reveal very strong visible and infrared light absorption. The B-TiO_2_ nanoparticles have oxygen vacancies and no Ti^3+^ ions. We evaluated the effectiveness of the as-prepared B-TiO_2_ nanoparticles and the feasibility of applying them to photocatalytic degradation tests. Very promisingly, the resultant B-TiO_2_ nanoparticles showed superior photocatalytic activity, far beyond that of commercial white TiO_2_ nanoparticles. 

## 2. Experimental

### 2.1. Materials 

Commercial catalyst white titanium dioxide nanoparticles (20–30 nm) and absolute ethanol were purchased from Macklin Biochemical. All the chemicals used in this study were analytical grade and were used as received. All the solutions were made using ultrapure Milli-Q (Millipore Corporation, Burlington, MA, USA) water.

### 2.2. Synthesis of B-TiO_2_ Nanoparticles

In a simple synthetic procedure, commercially available (W-TiO_2_) nanoparticles were used as precursors. W-TiO_2_ nanoparticles (0.5 g) and absolute ethanol (10 mL) were mixed in a 50 mL quartz tube using a magnetic stirrer to form a milky-white suspension. The milky-white suspension was stirred for 10 min. Then the suspension was poured into a sintering boat (length 6 cm, width 3 cm, height 1.5 cm) and transferred to a vacuum tube furnace (SK-G06163, Φ60/50 × 1000 mm). The suspension was annealed at 400 °C for 3 h. The detailed annealing procedure is as follows: The initial temperature is set to 50 °C and the temperature is raised at a rate of 5°C/min until reaches 400 °C and the temperature is maintained at 400 °C for three hours. Then, the temperature is lowered at a rate of 5 °C/min until reaches 50 °C. Finally, the sample was allowed to cool to room temperature and a black mass of B-TiO_2_ nanoparticles was obtained.

### 2.3. Characterization

The X-ray diffraction patterns (XRD) of the samples were obtained at room temperature using Cu Kα irradiation (λ = 1.5406 Å) operating at 36 kV tube voltage and 20 mA tube current. Data were collected between 10° and 70° (2θ) with a 0.02° step size. Raman spectra were recorded on a UV laser Raman spectrometer at λ = 785 nm. UV-vis diffuse reflectance (DRUV-vis) absorption spectra were recorded on an UV-2700 Spectrophotometer in the range of 200–800 nm. X-ray photoelectron spectra (XPS) were measured on an ESCALAB 250Xi using the reference of C1s (284.8 eV) with an excitation source of 150 W Al Kα (hv = 1486.6 eV) X-rays. The electron paramagnetic resonance (EPR) spectra were obtained using a JES-FA200 spectrometer (JEOL, Tokyo, Japan) at room temperature and an FEI Tecnai G2 F20 (FEI, Hillsboro, OR, USA) model was used to determine the size and morphologies of the nanoparticles. Sample specimens for the transmission electron microscopy (TEM) measurement were prepared by briefly ultrasonicating the sample powders in ethanol, followed by placing a drop of the suspension onto lacey support films that were dried before imaging. Fourier transformed infra-red (FTIR, 80/80v Bruker TENSOR27, BRUKER OPTICS, Karlsruhe, Germany) was used to analyze the functional groups in the catalyst from 500 to 4000 cm^−1^. Photoluminescence (PL) emission spectrum was recorded on an FLS980 fluorescence spectrophotometer equipped with a 450 W xenon lamp as the excitation source, at room temperature (excitation wavelength λ_ex_ = 300 nm).

### 2.4. Photocatalytic Test

Rhodamine B (RhB) and methylene blue (MB) are organic dye with a bright red color and blue color, respectively. They are widely applied as a test model pollutant in photocatalytic processes. Therefore, the photocatalytic activity of the two prepared samples was evaluated by monitoring the decomposition of (RhB)/(MB) in an aqueous solution under UV irradiation from a 500 W Hg lamp. The lamp was positioned in a cylindrical Pyrex vessel and cooled by circulating water to control the reaction temperature at about 27 °C. A quartz tube was used as the photo catalytic reactor. The sample of TiO_2_ (0.01 g) was mixed with an aqueous solution of RhB/MB (40 mL, 4 × 10^–5^ M). After stirring for 30 min in the dark, to reach an adsorption equilibrium between the photocatalyst and the RhB/MB solution, the mixture was exposed to UV light. Vigorous magnetic stirring was maintained in order to keep the B-TiO_2_ and W-TiO_2_ nanoparticles suspended in the RhB/MB solution. The concentration of aqueous RhB/ MB was determined with a UV-Vis spectrophotometer by measuring the peak intensity at 553/668 nm, respectively. The percentage of degradation was recorded as C/C_0_, where C and C_0_ refer to the absorbance of the RhB/MB solution after a certain time interval (20 min) and the initial absorbance, respectively.

The photocatalytic activity of B-TiO_2_ and W-TiO_2_ under visible light was also evaluated by monitoring the decomposition of RhB/MB. The apparatus for studying the photocatalytic decomposition of RhB/MB was identical under visible and ultraviolet light, except that an 800W Xe lamp was used instead of a Hg lamp and ultraviolet cut off filter (>420 nm) was applied to cut off the UV light. The amount of catalyst has also been increased to 0.02 g.

## 3. Results and Discussion

### 3.1. Characterization

**Figure 1:**
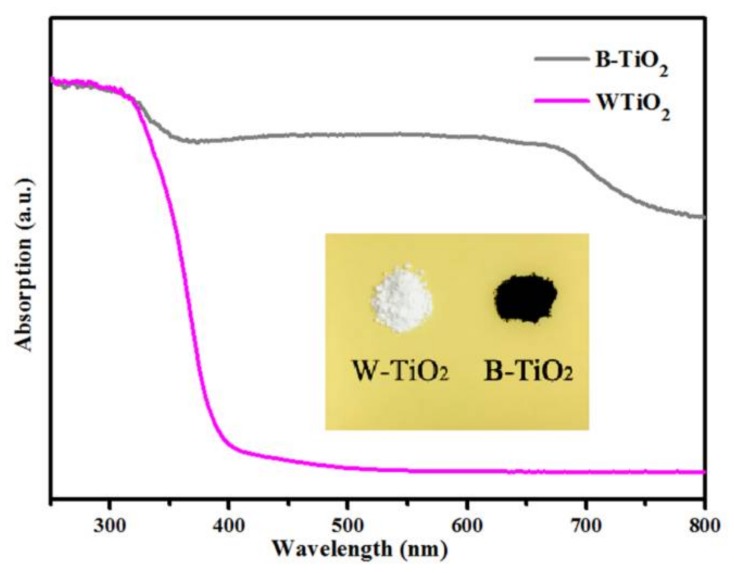
UV-Vis absorption spectra of black B-TiO_2_ and W-TiO_2_ nanoparticles. The insert is a photograph of B-TiO_2_ and W-TiO_2_ nanoparticles.

The prepared B-TiO_2_ nanoparticles showed an outstanding stability. No color change was observed for the B-TiO_2_ nanoparticles over one year after they were synthesized and stayed black even when annealed at 200 °C in air for 3 days. Figure 1 shows the UV-visible absorption spectra of the B-TiO_2_ and W-TiO_2_ nanoparticles. A large absorption peak is observed, for both, at wavelengths shorter than 400 nm (~3.1 eV), which can be attributed to the intrinsic bandgap absorption of crystalline anatase TiO_2_. Compared with pristine W-TiO_2_, the B-TiO_2_ extended the absorption from UV light to the visible and infrared light regions. The extended absorption is consistent with the changes of sample color from white to black (photograph shown in the inset of Figure 1). Furthermore, for the B-TiO_2_ nanoparticles, a step appeared at approximately 740 nm (~1.67 eV). This step could be due to oxygen vacancies with a trapped electron and it is discussed in detail in the EPR-analysis section.

**Figure 2:**
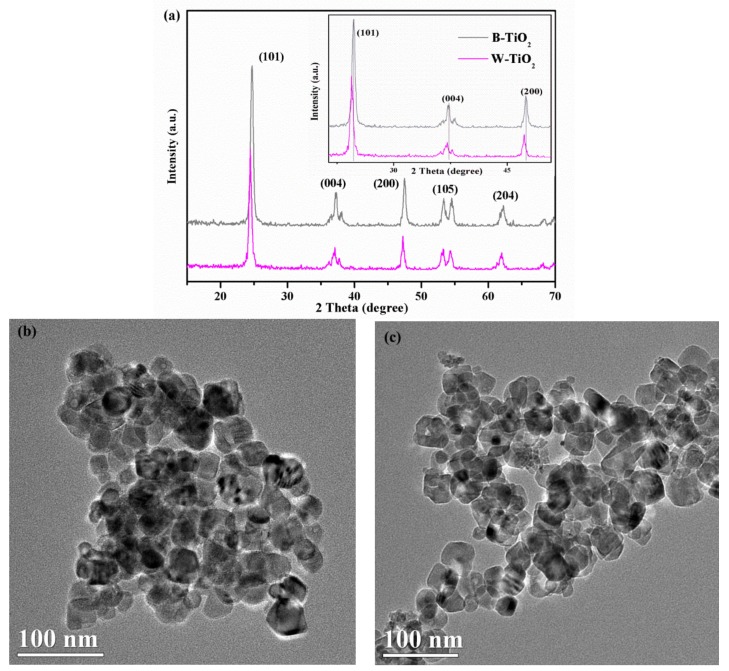

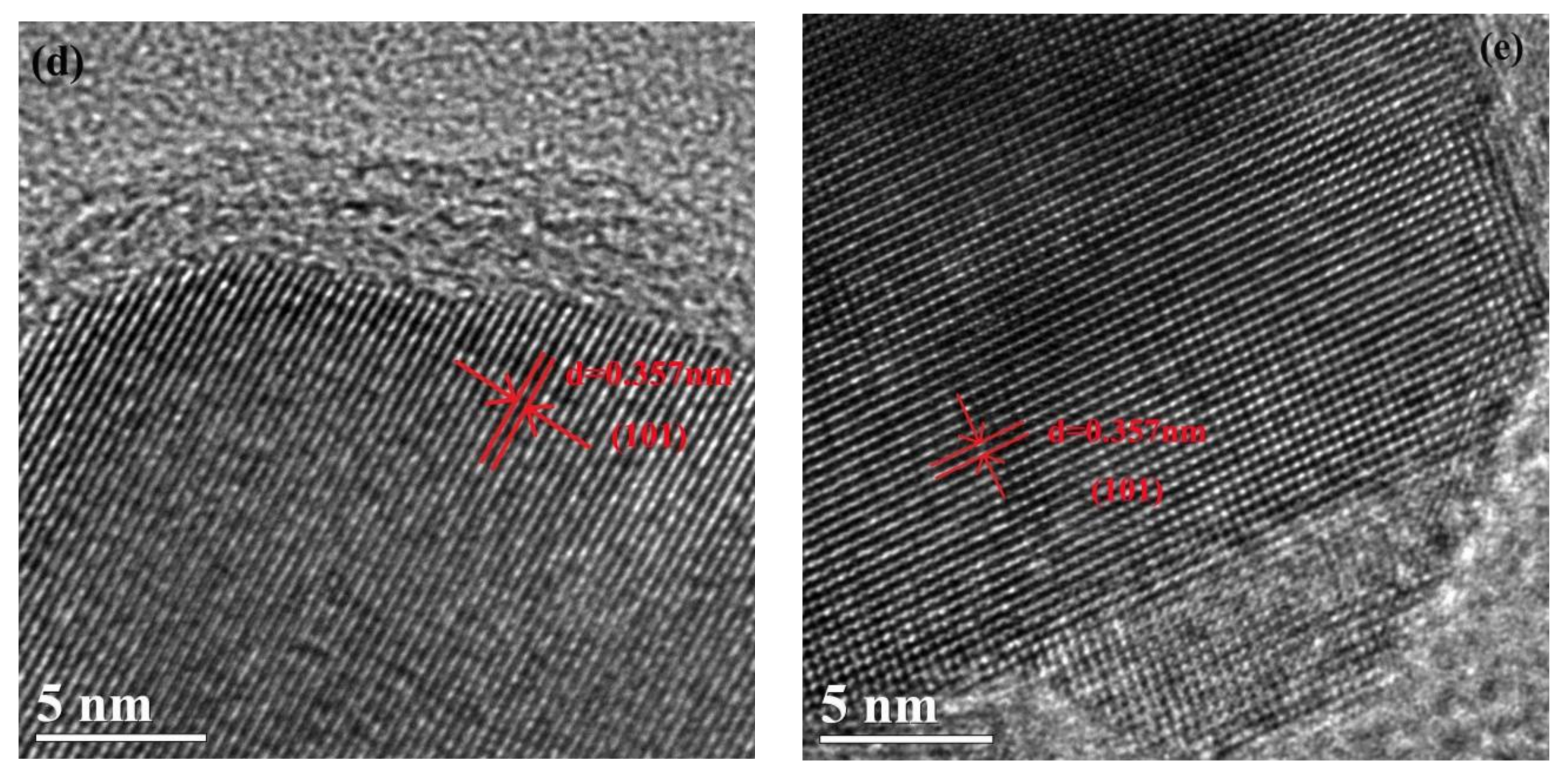
The XRD patterns of the as-prepared samples (**a**); TEM images of TiO_2_ nanoparticles before (**b**) W-TiO_2_ and after (**c**) B-TiO_2_ low temperature annealing; HRTEM images of TiO_2_ nanoparticles before (**d**) W-TiO_2_ and after (**e**) B-TiO_2_ low temperature annealing.

The crystalline structures of the B-TiO_2_ and W-TiO_2_ nanoparticles were determined by XRD analysis. The powder XRD patterns of the two samples, shown in Figure 2a, exhibited characteristic diffraction peaks matching the (101), (004), (200), (105) and (204) planes of anatase TiO_2_ (JCPDS Card No. 21-1272). It could also be seen that there was almost no phase change in the B-TiO_2_ nanoparticles and no other diffraction peaks could be observed after the low temperature annealing. However, from the local enlargement of diffraction peaks (inset of Figure 2a), the diffraction peaks of the B-TiO_2_ nanoparticles moved slightly to a higher angle. According to the Bragg equation (2dsinθ = λ, where d is the crystal spacing, θ the diffraction angle and λ the X-ray wavelength), the shift of peaks towards higher angles suggests that the lattice parameters decrease. This result can be explained in terms of the oxygen vacancies produced by the annealing of the samples [33].

The morphology and crystal structure of the B-TiO_2_ and W-TiO_2_ nanoparticles were examined by high-resolution transmittance microscopy (HR-TEM) analysis, as shown in Figure 2b–e. The TEM images showed similar particle sizes and morphologies for B-TiO_2_ (Figure 2b) and W-TiO_2_ (Figure 2c), which proves that the low temperature annealing did not change its morphology. The lattice fringe pattern with a spacing of ~0.35 nm, shown in the high resolution TEM (HRTEM) images (Figure 2d,e), confirms the (101) plane of anatase TiO_2_ for the W-TiO_2_ and B-TiO_2_ nanoparticles (in agreement with the XRD analysis). Both the W-TiO_2_ and B-TiO_2_ nanoparticles were highly crystallized, as seen from the well resolved lattice features. However, other researchers have reported a disordered surface layer surrounding the crystalline core in the hydrogenated/aluminum-reduced black TiO_2_ nanoparticles obtained under high temperature (>450 °C) and high pressure (>5 bar) [22,34]. In contrast, the B-TiO_2_ nanoparticles prepared in this work used low temperature (400 °C) and atmospheric pressure conditions that did not lead to the specific core/shell structure consisting of a disordered surface layer and a crystalline core. The specific surface areas using the Barrett-Emmett-Teller (BET) technique were 52.8 and 61.9 m^2^/g for the B-TiO_2_ and W-TiO_2_ nanoparticles, respectively. The similar BET specific surface areas confirmed that the crystal size of the B-TiO_2_ nanoparticles did not increase with the low temperature annealing, consistent with the above-discussed TEM results. 

**Figure 3:**
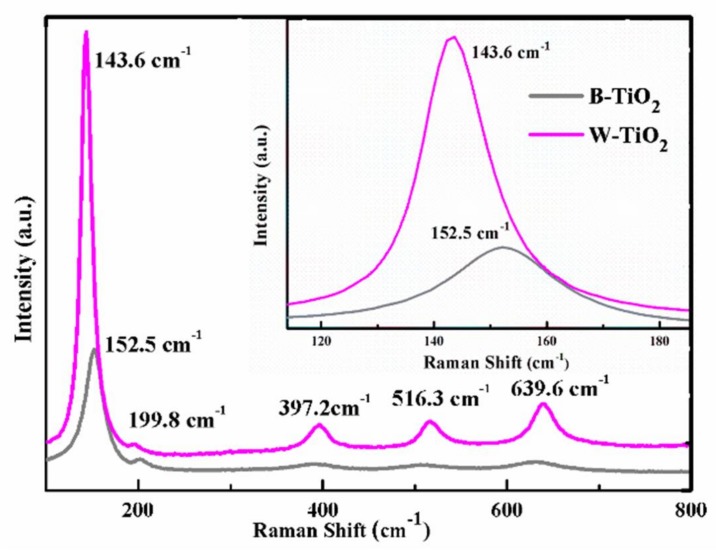
Raman spectra of B-TiO_2_ nanoparticles and W-TiO_2_, inset is the magnification of E_g_ peak.

Raman spectroscopy is very sensitive to short-range distortions arising from microstructural defects. The structural characteristics of the B-TiO_2_ nanoparticles were further examined by Raman scattering, as shown in Figure 3. For the W-TiO_2_ nanoparticles, five characteristic Raman peaks at 143.6, 199.8, 397.2, 516.3 and 639.6 cm^−1^ were assigned as the E_g_, E_g_, B_1g_, A_1g_ + B_1g_ and E_g_ modes of the anatase phase, respectively [35]. However, compared to the W-TiO_2_ nanoparticles, it is clear that the frequency of the strongest E_g_ mode (at 143.6 cm^−1^) in the B-TiO_2_ nanoparticles, which results from the external vibration of the Ti-O bonds, had a large blue shift to 152.5 cm^−1^, accompanied by peak broadening. As reported in previous studies, ruling out grain size effects, the blue shift and peak broadening of the anatase phase TiO_2_ can be ascribed to the oxygen stoichiometry [36]. Since the B-TiO_2_ and W-TiO_2_ nanoparticles had similar particle sizes, the blue shift and broadening of the strongest mode at 143.6 cm^−1^, can be attributed to a shortening of the correlation length due to the presence of oxygen vacancies (nonstoichiometric). Thus, Raman spectroscopy unambiguously supported the oxygen deficiency of the B-TiO_2_ nanoparticles. 

**Figure 4:**
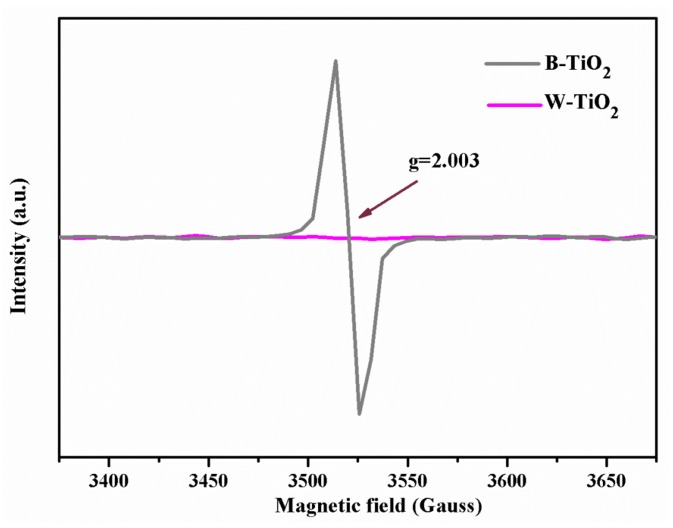
EPR spectra of B-TiO_2_ and W-TiO_2_ nanoparticles.

The existence of oxygen vacancies in the samples was also verified by electron paramagnetic resonance (EPR) at room temperature. EPR is widely-used to examine unpaired spins. In Figure 4, it is easily seen a strong EPR signal in the B-TiO_2_ nanoparticles at g = 2.003, which is characteristic of oxygen vacancies with a trapped electron in TiO_2_ and not of Ti^3+^ ions (g = 1.96–1.99) and consistent with the reported oxygen-deficient TiO_2−x_ [37,38]. Upon removal of an oxygen atom, one or two electrons will be localized in an oxygen vacancy site (reaction 1) and form color centers. The color centers associated with oxygen vacancies in TiO_2_ are the F, F^+^ and F^++^ centers (reactions 2–4) [39]. In our samples, one electron is localized in an oxygen vacancy state (F^+^-centers).

−O^2−^→ O + V_O_ + 2e^−^ (1)

V_O_ + 2e^−^→ F (2)

V_O_ + 1e^−^→ F^+^ (3)

V_O_ + 0e^−^→ F^++^ (4)

Furthermore, Chen et al. have proved that electrons transit from the color center energy level to the conduction band bottom. The calculated excited energy of the F^+^-center electron is 1.67 eV [40], which is in reasonable agreement with the step value which appeared at approximately 740 nm (~1.67 eV) in the UV-Vis absorption spectra (Figure 1). Thus, the absorption spectral features in the 1.67 eV region (740 nm) are assigned to a transition from the ground state of the F^+^ center to its corresponding excited F^+*^ state. By contrast, the W-TiO_2_ nanoparticles did not contain any paramagnetic site, as a flat line was observed for this sample. Thus, the strong EPR signal peak and also the blue shift of the Raman peak, indicated that B-TiO_2_ nanoparticles had a high concentration of oxygen vacancies and that no Ti^3+^ ions existed. 

**Figure 5:**
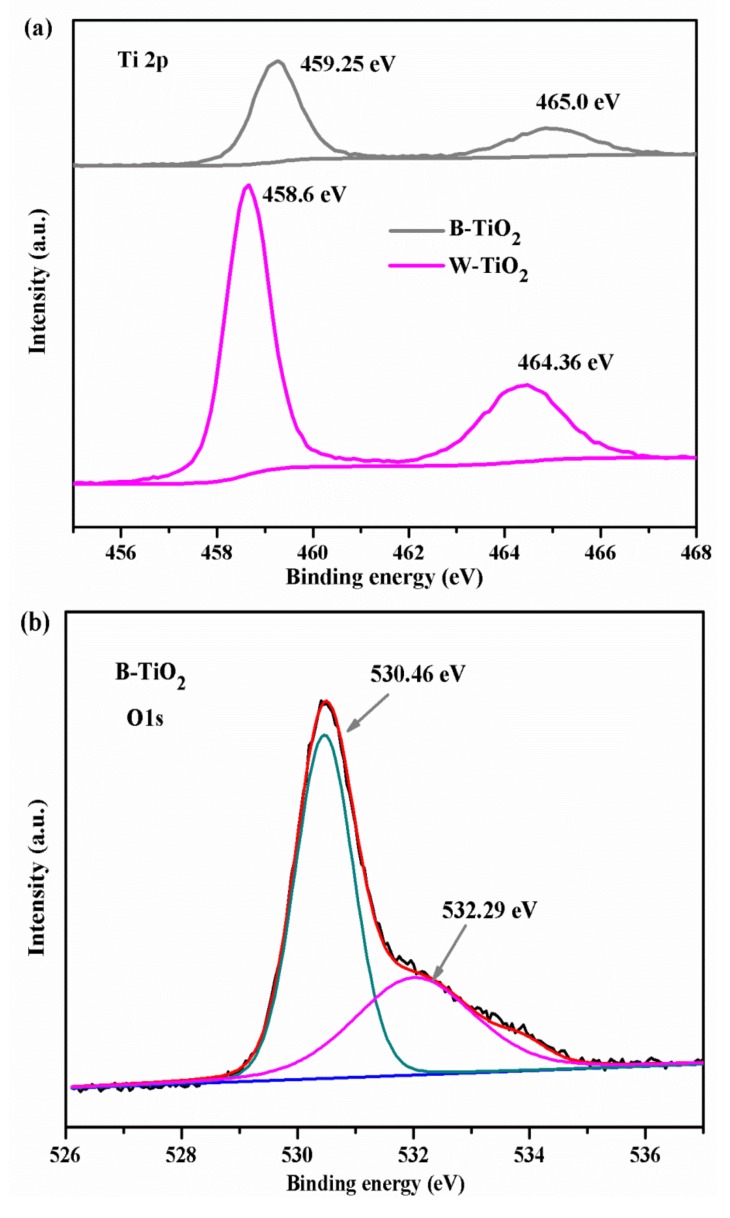

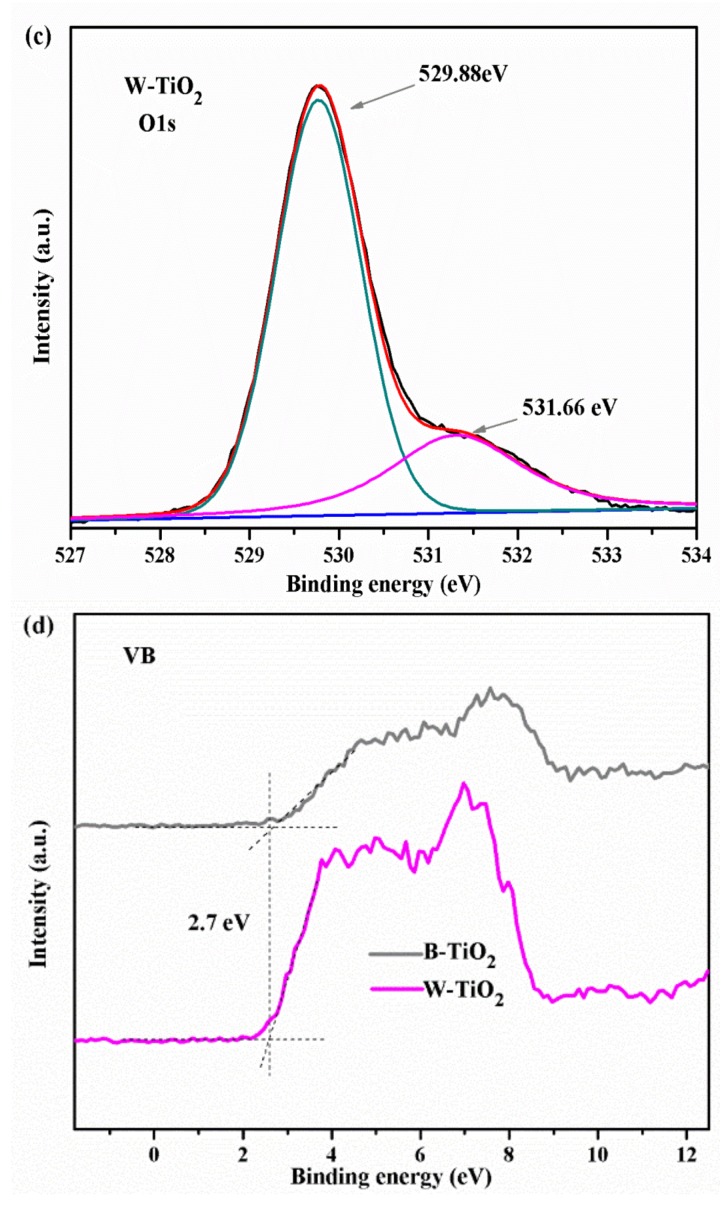
(**a**) The Ti 2p XPS spectra for the as-prepared B-TiO_2_ and W-TiO_2_ nanoparticles; (**b**)The O 1s XPS spectra for W-TiO_2_ nanoparticles and (**c**) the O 1s XPS spectra as-prepared B-TiO_2_ nanoparticles along with the Gaussian fits; (**d**) Valence-band XPS spectra of B-TiO_2_ and W-TiO_2_ nanoparticles.

X-ray photo-electron spectroscopy (XPS) analysis was conducted to investigate the chemical states of the Ti and O elements, to gain further insight into the oxygen vacancies in the B-TiO_2_ nanoparticles and the valence band position on the sample surface. Figure 5a shows the Ti 2p XPS of the B-TiO_2_ and W-TiO_2_ nanoparticles. For the W-TiO_2_ nanoparticles, the Ti 2p3/2 and 2p1/2 XPS peaks were centered at binding energies (BE) 458.6 and 464.36 eV and the calculated ΔBE value between Ti 2p1/2 and Ti 2p3/2 (ΔBE = BE(Ti 2p1/2) − BE(Ti 2p3/2)) was 5.76 eV. These features are typical for the Ti^4+^-O bonds in TiO_2_ [18]. The Ti 2p3/2 and 2p1/2 XPS peaks of the B-TiO_2_ nanoparticles were located at 459.25 and 465.0 eV, respectively, showing a BE positive shift of about 0.65 eV compared with the peaks of the W-TiO_2_ nanoparticles. However, the ΔBE value between Ti 2p1/2 and Ti 2p3/2 was 5.75 eV, indicating a normal state of Ti^4+^ in the B-TiO_2_ nanoparticles. According to previous papers on black TiO_2_, when the valence of Ti changes to Ti^3+^, additional peaks at lower binding energies can be observed, or the peaks will show a negative shift in binding energy [41,42].Our results confirmed that the charge states of the Ti atoms at the surface of the B-TiO_2_ sample were Ti^4+^ and that no Ti^3+^ existed at the surface. Additionally, the same phenomenon appeared in the O 1s XPS spectra. Figure 5b shows the high-resolution O 1s XPS spectra of the W-TiO_2_ nanoparticles: there are two peaks located at about 529.88 and 531.66 eV, which were attributed to the Ti^4+^-O bond and to surface hydroxyl groups (Ti-OH bonds), respectively. Compared with the W-TiO_2_ nanoparticles, the O 1s XPS peak of the B-TiO_2_ nanoparticles (Figure 5c) located at ~530.54 eV, also showed a clear positive shift in binding energy of about 0.66 eV, which is consistent with the Ti 2p. The extra peak existing in the O 1s spectra of the B-TiO_2_ nanoparticles at higher BE (~532.91 eV) was ascribed to surface free −OH species [43]. Furthermore, the calculated value ΔBE = BE(O 1s) − BE(Ti 2p3/2) = 530.54 eV − 459.25 eV = 71.29 eV, is close to that of anatase (71.4 eV) and quite different from that of the characteristic Ti^3+^-containing oxides (72.9–73.1 eV) [44]. These features indicated that the valence state of Ti did not change in the B-TiO_2_ nanoparticles. Reasonably, the clear positive shifts of binding energies of Ti 2p and O 1s were ascribed to the strong interaction between Ti^4+^ and oxygen vacancies, revealing lattice distortions on the B-TiO_2_ nanoparticles. It is possible that the oxygen vacancies, sustaining a positive charge, rejected the Ti atoms toward the adjacent oxygen atoms in the crystal structure of B-TiO_2_, consequently reducing the Ti-O bond length as well as increasing the BE of Ti 2p and O 1s [45,46]. Therefore, the positive shift of the XPS peak is due to the shortening of the correlation length because of the presence of oxygen vacancies. The valence-band maxima were estimated by a linear extrapolation of the peaks to the baselines, which gave a band edge position of 2.7 eV below the Fermi energy for both the B-TiO_2_ and the W-TiO_2_ nanoparticles, as shown in Figure 5d. Thus, the oxygen vacancies had a negligible effect on the valence-band position of the B-TiO_2_ nanoparticles surface. 

**Figure 6:**
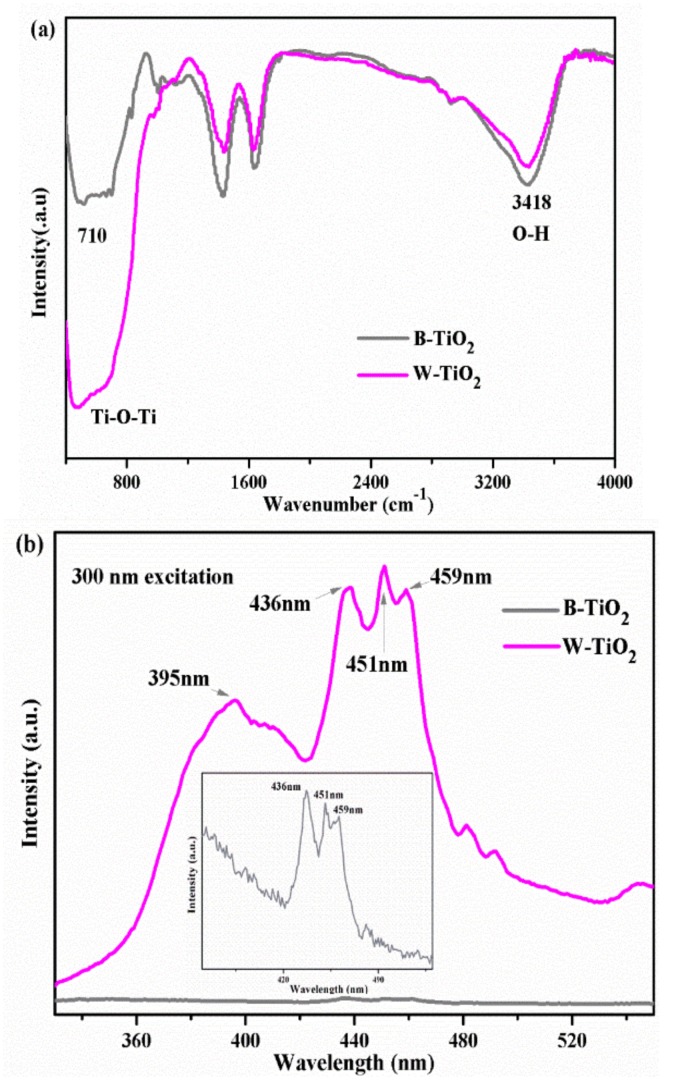
(**a**) FT-IR spectra of B-TiO_2_ and W-TiO_2_ nanoparticles; (**b**) Photoluminescence spectra of B-TiO_2_ and W-TiO_2_ nanoparticles.

To gain further insights on the chemical changes produced in the B-TiO_2_ nanoparticles, Fourier transform infrared (FTIR) spectroscopic measurements were performed, as shown in Figure 6a. Both the B-TiO_2_ and W-TiO_2_ nanoparticles showed similar absorption features from 500 cm^−1^ to 4000 cm^−1^. Characteristic features of the spectrum are the presence of a band at ~710 cm^−1^, due to the symmetric stretching vibrations of the Ti-O-Ti bonds of TiO_2_ and of a peak at about ~3418 cm^−1^, attributed to the stretching vibration mode of hydroxyls and adsorbed water [47]. Compared with the W-TiO_2_ nanoparticles, the infrared absorption of the B-TiO_2_ nanoparticles decreased greatly at ~710 cm^−1^. This change can be attributed to the presence of a high concentration of oxygen vacancies in the B-TiO_2_ nanoparticles [48].

The photoluminescence (PL) spectra of the B-TiO_2_ and W-TiO_2_ nanoparticles were assessed to investigate the behavior of the electron-hole recombination, which are very important for the photocatalytic activity. Their emission spectra, in the wavelength range of 320–560 nm with excitation at 300 nm, are shown in Figure 6b and were very similar. The main emission peaks of the W-TiO_2_ nanoparticles appeared at 395 nm (3.1 eV), 436 nm (2.8 eV), 451 nm (2.75 eV) and 459 nm (2.7 eV), respectively. The first one is attributed to the bandgap transition, corresponding to the bandgap energy of anatase. The peaks at 451 nm and 459 nm were attributed to band-edge free excitons [47]. The PL peak intensities of the B-TiO_2_ nanoparticles showed a significant decrease compared with those of the W-TiO_2_ nanoparticles. Furthermore, the main emission peak at 395 nm (3.1 eV), could not be observed, which indicated that the recombination rate of photo-generated electrons and holes had been considerably inhibited in the B-TiO_2_ nanoparticles [49]. Given the fact that oxygen vacancies usually serve as electron traps, the weaker intensity of emission and disappearance of the emission peak at 395 nm should be due to the increased oxygen vacancies. All the results indicated that the B-TiO_2_ nanoparticles had a relative low recombination rate of electrons and holes. Normally, the low recombination rate of electrons and holes favors high photocatalytic activity. 

### 3.2. Photocatalytic Activity

**Figure 7:**
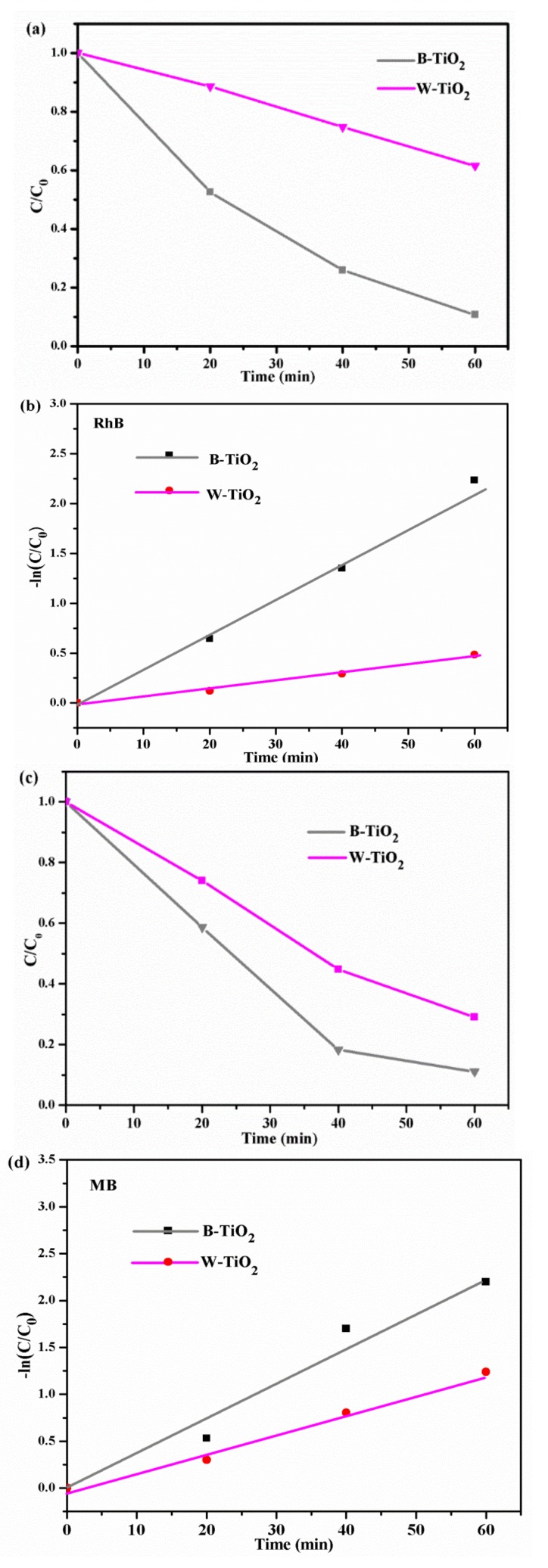

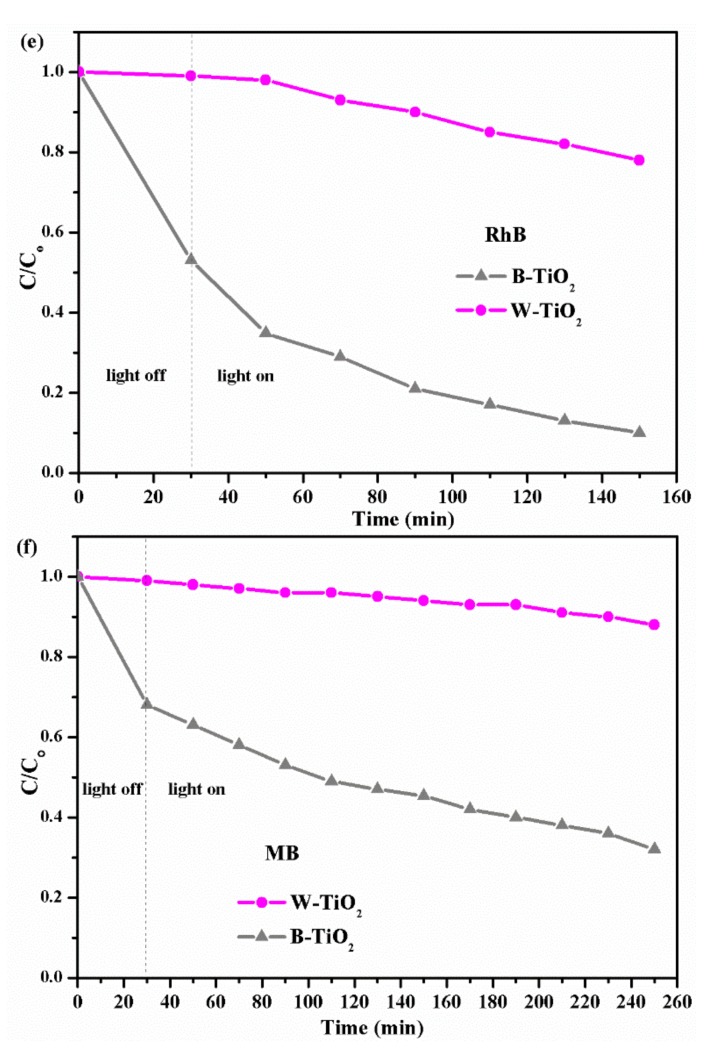
Comparison of photodecomposition of RhB (**a**) and MB (**c**) with different photocatalysts under UV light. −ln(C/C_0_) of the RhB (**b**) and MB (**d**) concentration as a function of UV light irradiation time. Photodecomposition of RhB (**e**) and MB (**f**) with different photocatalysts under visible light.

The photocatalytic activities of the as-prepared samples were evaluated by monitoring the decomposition of RhB in an aqueous solution under UV light irradiation. Commercial W-TiO_2_ nanoparticles were used as the reference photocatalyst. Before the irradiation of these samples, an adsorption experiment was performed in the dark in order to ensure the adsorption equilibrium of RhB and MB on the catalyst surface. As shown in Figure 7a, the B-TiO_2_ nanoparticles distinctly showed much better photocatalytic activity than the W-TiO_2_ nanoparticles. After 20 min of UV-light irradiation, the B-TiO_2_ nanoparticles could decompose about 50% of the RhB dye, while the value for the W-TiO_2_ nanoparticles was very low (~10%). When the irradiation time was prolonged to 60 min, the RhB dye was almost completely decomposed (96.2%) by the B-TiO_2_ nanoparticles but the W-TiO_2_ nanoparticles only decomposed about 40%. The Figure 7a also shows that the photocatalytic decomposition of RhB, for the two samples, followed a pseudo-first-order reaction [50]; the pseudo-first-order kinetics of the decompositions are illustrated in Figure 7b. The B-TiO_2_ nanoparticles showed a first-order rate constant of k_app_ = 0.036 min^−1^, 4.5 times greater than that of the W-TiO_2_ nanoparticles (k_app_ = 0.008 min^−1^), under UV light irradiation.

The UV-light photocatalytic activity of the B-TiO_2_ nanoparticles was further evaluated by measuring the photo-catalytic-degradation efficiency of the MB dye in aqueous solution. As shown in Figure 7c, under UV-light irradiation, in the short period of 20 min, the B-TiO_2_ nanoparticles could decomposed about 40% of the original organic MB dye. After 60 min irradiation, the MB dye had been almost completely decomposed (95.2%) by the B-TiO_2_ nanoparticles. In contrast, the W-TiO_2_ nanoparticles photocatalyst were able to decompose only about 70% of the original MB after 60 min irradiation. Figure 7d shows that the first-order rate constants of the B-TiO_2_ and W-TiO_2_ nanoparticles were 0.036 min^−1^ and 0.02 min^−1^, respectively. The as-prepared B-TiO_2_ nanoparticles had about 1.8 times better MB-decomposing photocatalytic activity than the W-TiO_2_ nanoparticles, under the same conditions.

The photocatalytic activity of the B-TiO_2_ sample was also investigated under visible light (λ > 420 nm) illumination, as shown in Figure 7e,f. Figure 7e shows the photodecomposition of RhB by B-TiO_2_ and W-TiO_2_ samples. Under visible light illumination, the RhB solutions containing the B-TiO_2_ undergo significant decomposition and became nearly transparent within 150 min. In contrast, W-TiO_2_ exhibits limited activity on photodecomposition of RhB solution. We also evaluated the decomposition of MB dye solution as a model reaction under visible-light irradiation, shown in Figure 7f. The photocatalytic efficiency of B-TiO_2_ under these conditions is 34%, which is higher than that of W-TiO_2_ (88%) after an irradiation time of 250 min. Thus, the B-TiO_2_ samples clearly exhibit improved photocatalytic activity.

### 3.3. Mechanism

**Figure 8:**
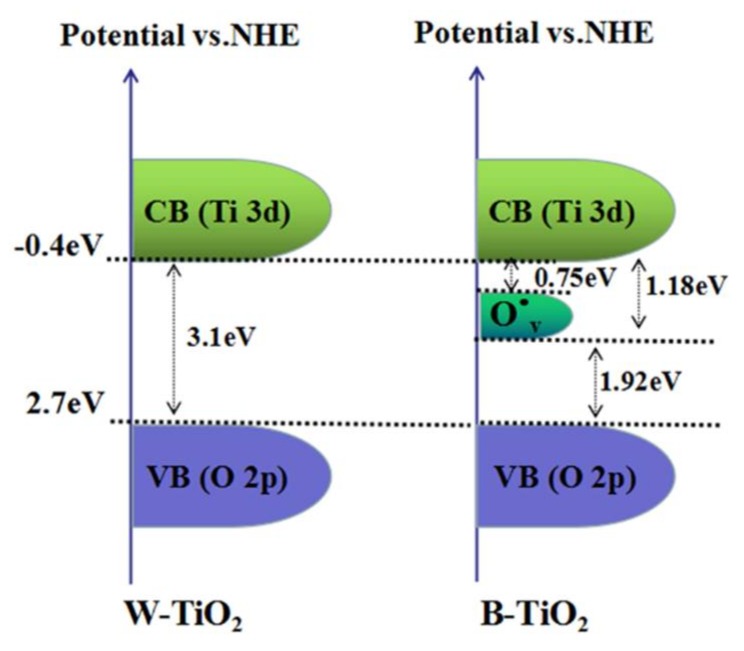
The band energy diagram of B-TiO_2_ and W-TiO_2_ nanoparticles.

In this work, the B-TiO_2_ nanoparticles greatly extended their absorption range from the UV- to visible- to infrared-light regions and their photocatalytic activity was greatly improved. All the characterization results proved that this B-TiO_2_ behavior is attributable to its high concentration of oxygen vacancies. The oxygen vacancies have been demonstrated to be electron donors in TiO_2_ [51] and have been considered to contribute to the enhanced donor density in B-TiO_2_ nanoparticles [27]. The electrons located on the oxygen vacancy states have a direct effect on the electronic structure of TiO_2_ by forming a donor level below the conduction band. The oxygen vacancies introduced localized states at 0.75–1.18 eV below the TiO_2_ conduction band minimum [46]. Consequently, the visible and near infrared-light absorptions are associated with transitions from the B-TiO_2_ valence band to the oxygen vacancy levels or from the oxygen vacancies to the TiO_2_ conduction band or with transitions from the ground state of the F^+^ center to its corresponding excited state F^+^*. In order to further understand our B-TiO_2_ nanoparticles, the energy band diagram of the B-TiO_2_ nanoparticles is schematized in Figure 8. The valence band maximum (VBM) and conduction band minimum (CBM) of TiO_2_ are derived mainly from the O 2p orbitals and the Ti 3d orbitals, respectively. According to the UV-vis absorbance spectra, the bandgap of both the B-TiO_2_ and the W-TiO_2_ nanoparticles was 3.1 eV. Furthermore, the increased oxygen vacancies can improve charge transport in TiO_2_ and shift its Fermi level toward the conduction band, facilitating the charge separation at the semiconductor/electrolyte interface [52,53]. The PL results also indicated that, in our experiments, the B-TiO_2_ nanoparticles had a low recombination rate of electrons and holes. A low recombination rate leads to a large increase in photocatalytic activity. 

## 4. Conclusions

The present work deals with the development of a facile method for the preparation of a highly active black TiO_2_ photocatalyst. The black TiO_2_ was prepared via low temperature annealing of commercial W-TiO_2_ catalyst nanoparticles. The prepared B-TiO_2_ nanoparticles showed remarkable photocatalytic activity for the degradation of the RhB and MB dyes. An appropriate amount of oxygen vacancies was introduced into the B-TiO_2_ nanoparticles and Ti^3+^ ions were not found. The oxygen vacancies are responsible for the increased visible-and infrared-light absorption as they can introduce localized states into the bandgap. At the same time, the oxygen vacancies act as traps for reducing the recombination of electrons and holes and significantly improve the e–h separation efficiency, thus greatly enhancing the photocatalytic activity.
